# UHF RFID Temperature Sensor Tag Integrated into a Textile Yarn

**DOI:** 10.3390/s22030818

**Published:** 2022-01-21

**Authors:** Sofia Benouakta, Florin Doru Hutu, Yvan Duroc

**Affiliations:** 1 Univ Lyon, Université Claude Bernard Lyon 1, INSA Lyon, Ecole Centrale de Lyon, CNRS, Ampère, UMR5005, 69622 Villeurbanne, France; yvan.duroc@univ-lyon1.fr; 2 Univ Lyon, INSA Lyon, Inria, CITI, EA3720, 69621 Villeurbanne, France; florin-doru.hutu@insa-lyon.fr

**Keywords:** RFID sensor, RFID yarn, temperature sensor

## Abstract

This paper presents the design of an ultra high-frequency (UHF) radio frequency identification (RFID) sensor tag integrated into a textile yarn and manufactured using the E-Thread^®^ technology. The temperature detection concept is based on the modification of the impedance matching between RFID tag’s antenna and the chip. This modification is created by the change in the resistance of a thermistor integrated within the tag system due to a temperature variation. Moreover, in order to obtain an environment independent detection, a differential approach is proposed that avoids the use of a pre-calibration phase by the use of a reference tag. Experimental characterization demonstrates the RFID sensor’s potential of detecting a temperature variation or a temperature threshold between 25 and 70 °C through the variation of the transmitted differential activation power.

## 1. Introduction

Passive RFID is a technology that originally identified objects associated to a device called a ‘tag’ using the backscattering technique. In general, an RFID tag can be simplified as the association between an antenna (usually a dipole) and an integrated circuit (IC), which processes the signal originating from the reader. However, there exists another category of RFID tags that do not include an IC (i.e., chipless tags) and where the signal backscattered to the reader consists on a ‘signature’ defined by the antenna (i.e., simple reflecting element) properties such as geometry and materials. Due to the absence of any active electronics in chipless RFID tags, these structures have a great potential to be employed for sensing applications without the risk of damaging the IC, for example, in an environment with a high temperature level [1,2,3,4]. However, despite their advantages, the main drawback of chipless tags is the absence of a dedicated standard and above all a strong limitation of use in terms of read range due to the impact of the environment: each object being also a reflective object [5]. Today, RFID technology has considerably evolved and inspired from the chipless systems; RFID tags, operating in the ultra-high frequency (UHF) band are used for sensing applications enabling the detection of a physical or mechanical property such as temperature, humidity, or strain; this is called a RFID sensor tag.

In order to integrate a sensing functionality into a RFID tag, two main approaches have been developed [6]. The first one is to add a sensor to the tag: by connecting the sensor to the tag or by integrating the two circuits in the same system. Today, there are RFID chips on the market that integrate sensors in the same packaging, for instance the chip manufactured by AMS [7]. The second approach is to use a physical property of the tag itself to sense the information. For example, if the antenna substrate is sensitive to humidity (Kapton substrate), the characteristics of the assembly will vary, and it will be possible to detect the humidity variation [8]. This type of sensor is often less accurate than the first one but simpler, less expensive, and not at all energy consuming. 

More specifically, the use of UHF RFID tags seems to be a very promising avenue for the deployment of the wearable concept, especially when it comes to providing information about the wearer. Wearable technology consists in combining electronics and everyday objects and can be classified into four categories: (i) accessories, (ii) patches, (iii) implants, and (iv) smart textiles. The category “smart textiles” has attracted a lot of interest as it allows the combination of electronic devices and textile materials to create smart garments. In the context of RFID, the electronic device is a wireless communicating system consisting of the RFID tag (chip and antenna). The RFID tag must be associated with the garment and thus meet several constraints such as comfort for the user but also, for example, washability. These different application constraints depend strongly on the way the tag is associated with the garment and, consequently, on the manufacturing process. 

Many UHF RFID sensor tags have been developed using a wide range of fabrication techniques such as 3D printing for temperature sensing [9,10], inkjet printing [11], or embroidery [12] for moisture sensing. On the other hand, particularly well adapted to the wearable, there is a commercial UHF RFID textile thread [13] that is based on the patented E-Thread technology [14]. This technology consists in associating the antenna and the chip by an automatic assembly process in order to obtain a spool of textile thread integrating a series of RFID tags. One of the interests of this technique is to integrate the tag, here a simple wire, directly into the object as soon as it is manufactured. In this case, for the user, the intrusion of the tag into the garment is both invisible and without impact on comfort.

In the manufacturing process of the original E-Thread RFID tag, the first step is to modify the integrated circuit (IC) geometry. More precisely, the IC is modified in such a way that two lateral grooves are scraped in order to receive the antenna’s micro-wires. Then, the chip is assembled with the antenna wires, and this step is performed with an automated equipment, which is able to produce a continuous filament made of the two conductive wires together with the IC’s, which are assembled at a fixed interval. A resin is employed in order to mechanically reinforce the IC assembly. The final step is the textile packaging. 

The objective of the presented work is to add a temperature sensor functionality to this UHF RFID yarn and thus transform it into a sensor tag while keeping the passive nature of the tag and respecting the constraints of the manufacturing process based on the E-Thread technology. The proposed idea is to add in the impedance matching loop a thermistor of which the resistance variations according to the temperature could be detected and thus exploited for a temperature estimation. A first proof of concept was presented in [15], showing that the reading range is varied from 4.2 to 6.2 m for a temperature variation from 25 to $70{\;}^{{}^{\circ}}C$. Based on this study, in this paper, the principle of this new temperature sensor tag is recalled, and new results are presented. On the one hand, it is shown that the temperature can be deduced simply and directly from the activation power. However, for such a direct measurement, it is necessary to perform a preliminary calibration in order to consider the environment and to compensate for the variability of the activation power that depends on it. Moreover, this calibration would have to be repeated as soon as the propagation environment varies, making the use of such a measurement system rather constraining. To avoid the need for calibration, a reference tag is associated with the sensor tag, and the temperature measurement is then deduced differentially without prior calibration.

The rest of the paper is organized as follows. Section 2 presents the temperature sensor tag configuration and its theoretical concept. Based on the simulation results, Section 3 highlights the proposed principle by showing in particular the impact of the temperature variation on impedance matching. Section 4 presents the fabricated prototype, the differential power theory to avoid a calibration step, and the full experimental setup including the different steps of a differential measurement. Section 5 details and analyses the obtained experimental results and their limits with a comparison to other UHF RFID temperature sensor tags existing in the literature. Finally, Section 6 draws the conclusion and perspectives of this work.

## 2. Presentation of the RFID Yarn Including a Temperature Sensor

### 2.1. Principle of the Integration of the Sensing Capability in the RFID Yarn

**Figure 1:**
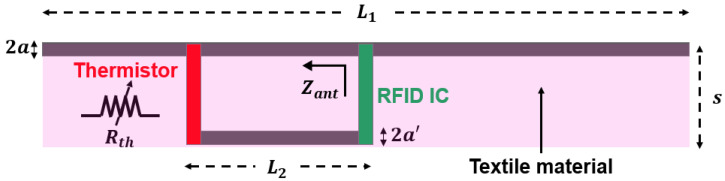
Configuration of the RFID temperature sensor tag integrated into a textile yarn.

The original passive UHF RFID yarn [13] consists of a dipole type antenna, more precisely a radiating element of length $L_{1}$ and radius a, associated to a Monza 6 chip [16]. The impedance matching between the tag’s integrated circuit (IC) and tag’s antenna is achieved by using a short-circuited stub of length $L_{2}$ and radius $a^{\prime }$ based on the gamma-matching principle. The radiating element and the short-circuited stub are separated by a distance s from center to center. As shown in Figure 1, the proposed idea is simply to replace the short circuit with a thermistor. Thus, a temperature variation that modifies the resistance $R_{th}$ of the thermistor will lead to an impedance mismatching.

### 2.2. Theoretical Concept of the Sensing Capability

The impedance mismatching of the tag [17], due to a temperature change, can be expressed through the power transmission coefficient τ given in (1) [18]:

(1) $$\tau =\frac{4R_{chip}\cdot R_{ant}}{{\left|Z_{chip}+Z_{ant}\right|}^{2}}\;$$

where $Z_{chip}=R_{chip}+jX_{chip}$ is the IC’s impedance and $Z_{ant}=R_{ant}+jX_{ant}$ is the antenna’s impedance. This coefficient, τ, whose maximum value is 1, characterizes the impedance matching between the antenna and the RFID chip (whose complex impedance varies with the frequency and the power absorbed by the chip), which directly impacts the tag’s read range.

The impedance of the loaded stub, seen at the center of the radiating element, is a function of the thermistor resistance,$\;R_{th}$, that can be expressed using the transmission line theory as:

(2) $$Z_{th}=Z_{0}\frac{R_{th}+jZ_{0}\tan\theta }{Z_{0}+jR_{th}\tan\theta }\;$$

where $\theta =2\pi L_{2}/\lambda$ is the electrical length of the stub (with the λ wavelength) and $Z_{0}$ is the characteristic impedance of the transmission line considered in free space. Specifically, the impedance $Z_{0}$ is the characteristic impedance of the two-wire transmission line with radii a and $a^{\prime }$ and separation s. Its expression is given in [19,20] and can be approximated [19] as follows:

(3) $$Z_{0}=60\;\cosh^{-1}\left(\frac{s^{2}-a^{2}-{a^{\prime }}^{2}}{2aa^{\prime }}\right)≅276\;\log_{10}(\frac{s}{\sqrt{aa^{\prime }}})$$

The input impedance of the antenna, $Z_{ant},$ is the result of a shunt between the impedance of the radiating dipole, $Z_{a},$ and the impedance of the loaded stub. It can be expressed as follows:

(4) $$Z_{ant}=\frac{{\left(1+\alpha \right)}^{2}Z_{a}Z_{th}}{{\left(1+\alpha \right)}^{2}Z_{a}+Z_{th}}$$

where ${\left(1+\alpha \right)}^{2}=(1+{\left(ln\left(s/a^{\prime }\right)/\left[ln\left(s/a^{\prime }\right)-ln\left(a/a^{\prime }\right)\right]\right)}^{2}$ is the step-up factor, which is equal to 4 when $a=a^{\prime }$. Note that as for any dipole antenna, $Z_{a}$ is depends on the frequency and is equal to 73+j42.5 Ω at the resonance frequency. Finally, it is also worth noting that when $R_{th}=0\;Ω$, the sensor tag is equivalent to the original RFID yarn with the short-circuit for the impedance matching circuit.

## 3. Design and Characterization in Simulation of the RFID Yarn Including a Temperature Sensing

### 3.1. Geometric Sizing and Parameterization

The RFID temperature sensor tag described in Section 2.1 has been designed using the CST Microwave Studio 3D electromagnetic simulation tool. Where the time domain solver has been used based on the Finite Integration Technique (FIT) with a hexahedral adaptive meshing [21]. The RFID temperature sensor tag dimensions, summarized in Table 1, have been optimized for operation in the European UHF RFID band. 

The surrounding textile material has been modeled as a dielectric material with a relative permittivity $\varepsilon_{r}=2$. The chosen thermistor is a negative temperature coefficient (NTC) thermistor [22], which is characterized by a decrease in its resistance for an increase in temperature.

### 3.2. Impact of a Temperature Variation on the Impedance Matching of the RFID Tag

In order to estimate the impact of the temperature change on the power transmission coefficient, the resistance of the thermistor is varied between two limiting values :0 Ω and 22 Ω, to reflect a decrease in temperature from 150 to 25 °C (corresponding to an average ambient temperature). As previously mentioned, for temperature values above 150 °C, the resistance of the thermistor is zero, which can be considered as a short-circuit: the impedance matching case is then achieved. In the RFID domain, this means that the impedance of the antenna is not equal to a reference impedance (typically 50 Ω) but is set by the chip. Specifically, it must be equal to the conjugate impedance of the input impedance of the chip in the energy scavenging state.

**Figure 2:**
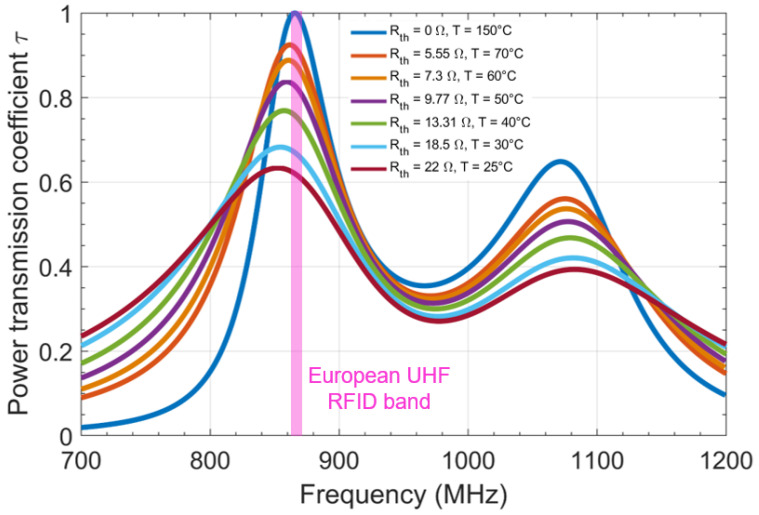
Impact of the thermistor’s resistance change due to temperature on the RFID sensor tag.

Figure 2 shows the variation of the tag’s power transmission coefficient, τ, as a function of the frequency considering several values of the thermistor resistance to simulate a temperature change ($R_{th}=0\;Ω;\;5.5\;Ω;\;7.30\;Ω;9.77\;Ω;\;13.31\;Ω;\;18.50\;Ω;22\;Ω$). First, it should be noted that the power transmission coefficient has two resonances that are the result of the two conductors present in the tag: the radiating element of length $L_{1}$ and the stub of length $L_{2},$ respectively. If we consider the first resonance (which is in the standard RFID band), we observe an impedance mismatching (i.e., the maximum value of the power transmission coefficient decreases) and at the same time a shift of the resonance frequency to lower frequencies. Moreover, if we now consider a fixed operating frequency (e.g., 868 MHz), then the variation of the transmission coefficient as a function of temperature can be exploited: this constitutes the principle of the proposed textile thread sensor.

## 4. Experimental Evaluation of the RFID Yarn Including a Temperature Sensor

### 4.1. Fabricated RFID Temperature Sensor Tag Yarn

**Figure 3:**
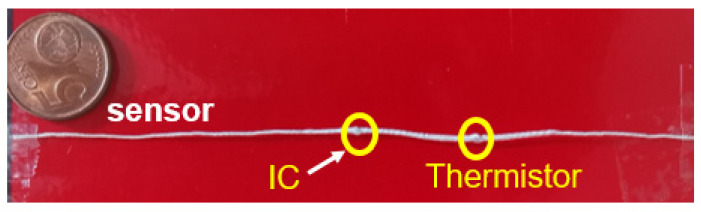
Fabricated temperature sensor tag using E-Thread technology.

The designed RFID temperature sensor tag yarn was fabricated using the E-Thread^®^ assembly process. Figure 3 shows a picture of a component of the RFID textile yarn spool; the short-circuit in the matching circuit was replaced by the thermistor. 

### 4.2. Temperature Measurement without Environmental Calibration: A Differential Approach

As shown in the simulation part (Section 3.2), a variation of temperature will imply a modification of the power transmission coefficient, and consequently a modification of the minimum activation power of the tag. However, the minimum activation power also depends on the propagation environment, especially in the vicinity of the tag. Additionally, if the propagation environment is modified, this power also varies, and it is no longer possible to detect if the observed change is actually related to the temperature or to any other parameter of the environment. Consequently, it would be necessary to carry out a calibration for a given fixed situation; and for each change of the environment to redo this calibration. To overcome this problem, an alternative is proposed by adding a reference tag (without detection capability) in the vicinity of the sensor tag. Assuming that the propagation channel (i.e., environment) of the two tags is similar, the reference tag will provide a reference minimum activation power that depends on the environment but not on the temperature. The comparison with the minimum activation power of the sensor tag will then allow to deduce the temperature variation. This comparison is simply the difference between the two minimum activation powers (with and without sensor) as detailed analytically below to deduce the so-called differential power. The differential technique has been employed in both chipless [23] and UHF RFID [24] sensor tags. 

In a general way, the transmitted power by the reader,$\;P_{0}$, can be written as follows [25]:

(5) $$P_{0}=P_{tag}-G_{t}-PL-\chi -G_{0}\tau_{0}\;\;\;\;\;\;\;\;\;\;\;\;\left(\mathrm{dBm}\right)$$

where $P_{tag}\;\left(\mathrm{dBm}\right)$ is the power received by the tag, $G_{t}$ (dBi) is the realized gain of the reader antenna, $G_{0}\;\left(\mathrm{dBi}\right)$ is the antenna gain of the tag, $\tau_{0}$ is the power transmission coefficient, PL (dB) is the propagation path loss, and χ (dB) is the polarization matching coefficient. 

Note that this expression is general and can therefore be used for the reference tag, which is performed here using the index 0 for clarity (i.e., the tag is the reference tag characterized by $G_{0}$ and $\tau_{0}$). Thus, (4) can be also rewritten for the sensor tag as follows:

(6) $$P_{t}=P_{tag}-G_{t}-PL-\chi -G_{tag}\tau \;\;\;\;\;\;\;\;\;\;\;\;\left(\mathrm{dBm}\right)$$

where only the term ‘Gτ’ is modified: in (6), $G_{tag}$ (dBi) is the antenna gain and τ is the power transmission coefficient, both corresponding to the sensor tag. At the RFID reader’s level, it is worth noting that the quantity, $P_{t}G_{t}$, expressed in linear, represents the equivalent isotropic radiated power (EIRP). Its maximum value depends on the regulations in force according to the geographical area of exploitation; for example, the value imposed by the European Telecommunications Standards Institute (ETSI) is 3.28 W. 

With the powers $P_{0}$ and $P_{t}$ that correspond to the minimum activation power for each of the tags (reference and sensor), the power difference, here called differential power, is necessary to activate, on the one hand, the reference tag and, on the other hand, the sensor tag will be an image of the temperature and given by the following expression:

(7) $$\Delta P=P_{t}-P_{0}=G_{0}\tau_{0}-G_{tag}\tau \;\;\;\;\;\;\;\;\;\;\;\;\left(\mathrm{dB}\right)$$

### 4.3. Experimental Setup

**Figure 4:**
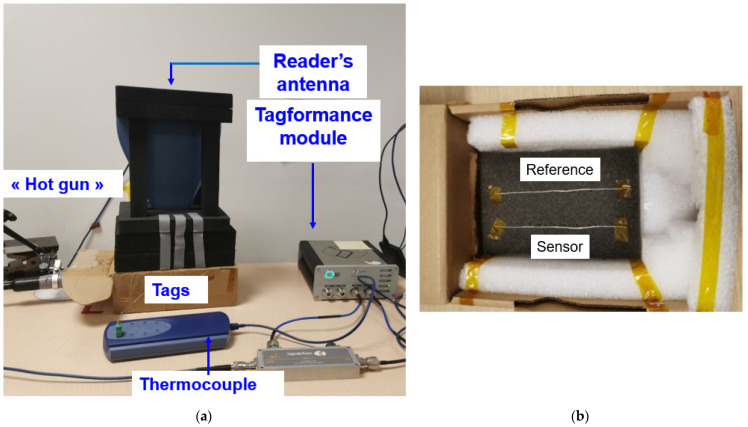
Experimental setup: (**a**) measurement setup elements; (**b**) RFID temperature sensor tag (at the bottom) and reference tag (at the top) placement environment.

The complete measurement protocol is presented in Figure 4. The reference tag and the temperature sensor tag are placed in a closed box as shown in Figure 4b. The two tags are aligned and separated by a distance of 4 cm. This distance is relatively small. It has been verified that it does not lead to a significant mutual coupling effect between the two tags. Moreover, it allows to assume that the propagation channels for each tag are similar.

The temperature inside the box containing the two tags is controlled with a hot air gun, and its temperature is measured with a thermocouple. The temperature range to which the tags are subjected varies from 25 to 70 °C. The maximum temperature was set at 70 °C in practice in order not to alter the tags. The RF measurements are performed using the Tagformance Pro bench [26] for the 800–1200 MHz frequency range. This bench allows to activate the tags according to the ISO 18000-6c protocol.

**Figure 5:**
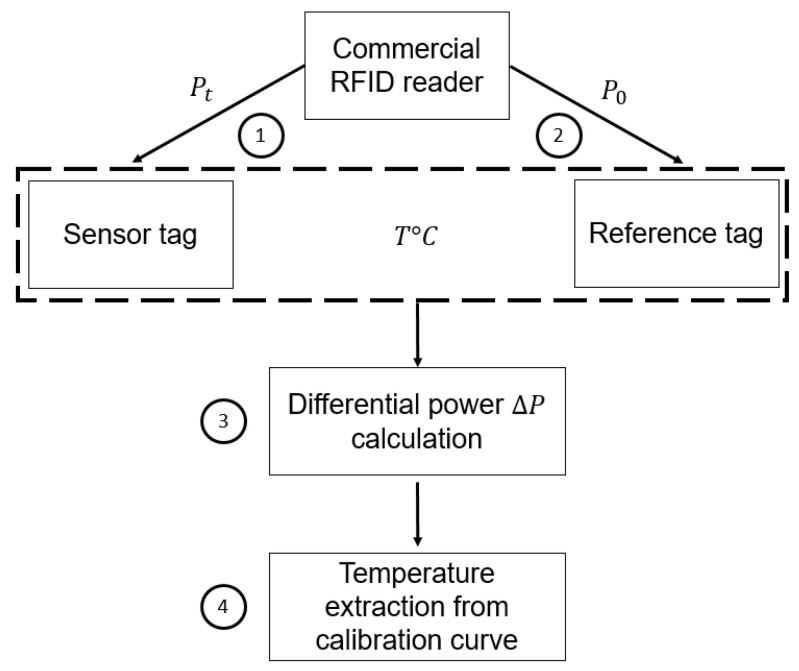
The proposed methodology of temperature extraction with a differential approach using a commercial RFID reader.

Using the described experimental setup here above, the differential power extraction can be summarized in few steps: *(i)* the reader’s transmitted power is gradually increased for the sensor tag and the reference tag one after another; *(ii)* the reading process is stopped once the IDs of the tags (sensor + reference) are returned and respective activation transmitted powers are extracted; *(iii)* the differential power ΔP is calculated for a given temperature. Finally, from the obtained result, by using a calibration curve, a temperature value can be attributed to every differential power. Figure 5 shows a diagram that summarizes the described protocol.

## 5. Measurement Results and Discussion

### 5.1. Impact of the Temperature Change on the Transmitted Differential Activation Power

**Figure 6:**
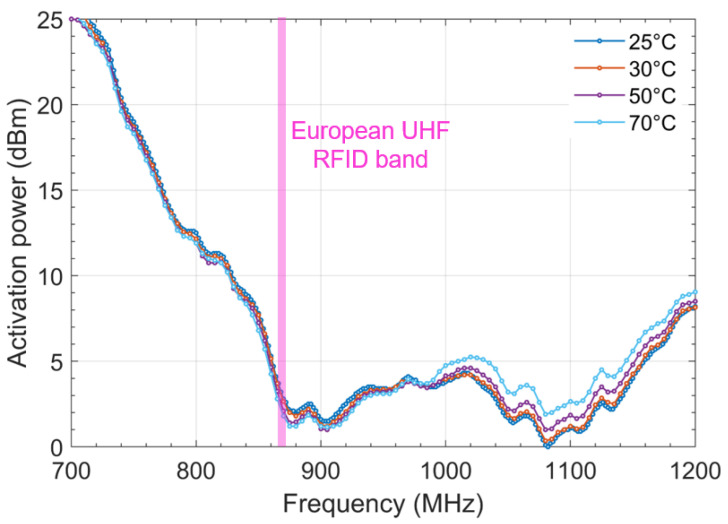
Impact of the temperature on the activation power of the reference tag.

**Figure 7:**
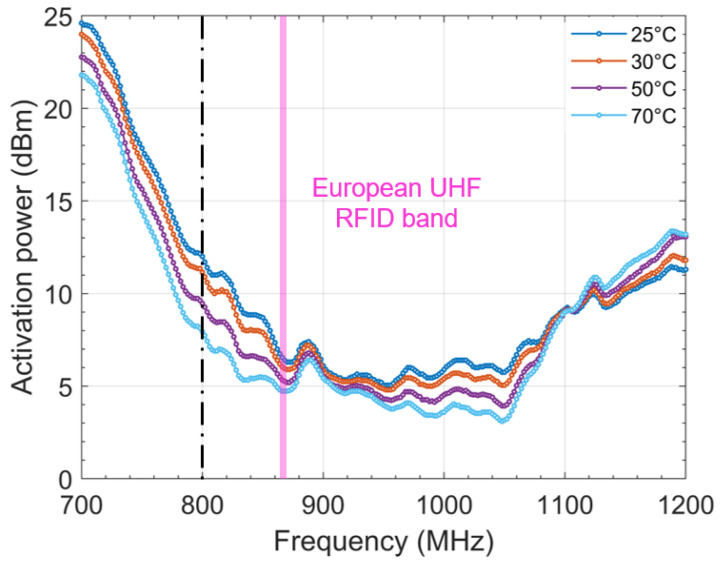
Impact of the temperature on the activation power of the sensor tag.

Figure 6 and Figure 7 show the activation power required for the reference tag and the sensor tag, respectively. The reference tag is practically not impacted by the temperature increase for the first resonance frequency (which is the frequency of interest). The temperature impacts the highest frequencies, which can be explained by the premise of expansion of the conductors when temperatures increase. However, this is of no consequence for the application and the considered frequency range. 

In the case of the sensor tag, as expected and detailed in the theoretical part, the minimum activation power decreases when the temperature increases. However, if the concept is experimentally validated, sensitivity could apparently be improved. Indeed, for a frequency around 800 MHz, the impact of the temperature variation is more accentuated.

**Figure 8:**
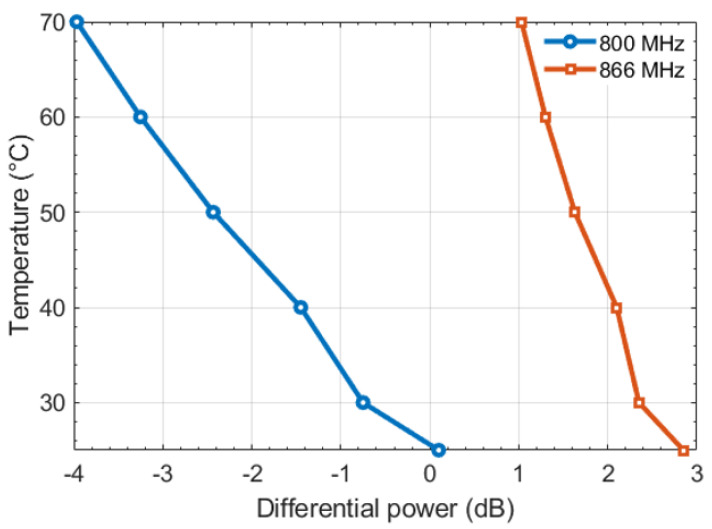
Temperature deducing using differential activation power method.

Figure 8 shows the variation of temperature versus differential power assuming two operating frequencies for illustration and proof of concept: 800 and 866 MHz. It can be observed that a decrease in the temperature value corresponds to an increase of the differential power level, which is coherent with (7). Moreover, a quasi-linear behavior of the temperature in the range of 40–70 °C can be noticed and corresponds to slopes (absolute value) of 11.4  and $33.3{\;}^{{}^{\circ}}C/\mathrm{dB}$ at the frequencies 800  and 866 MHz, respectively.

It is important to notice that the temperature value extraction depends strongly on the reader in terms of the transmitted power resolution. Consequently, the smaller the temperature slope, the easier it is to extract the temperature value. In the current state, it may be concluded that at 866 MHz, the sensor tag is more suited for a temperature threshold detection than for an exact temperature value. On the contrary, for an operating frequency of 800 MHz a temperature detection in terms of absolute values may be considered.

### 5.2. Impact of the Temperature Change on the Read Range

The method presented above allows an estimation of the temperature using an approach based on the differential activation power that can be applied using a commercial RFID reader. In the RFID context, the read range is an important criterion that characterizes the performance of the tag and is highly dependent on its impedance matching. It seemed interesting to evaluate the impact of the temperature variation on this quantity. 

**Figure 9:**
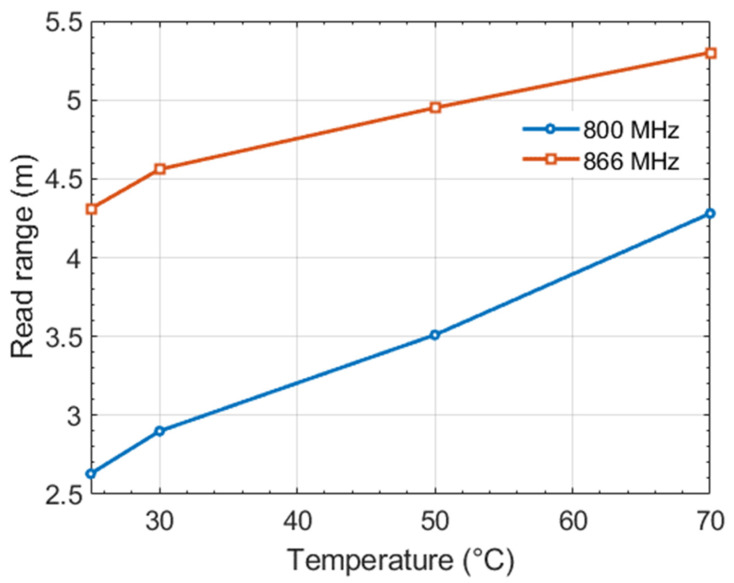
Impact of the temperature on the sensor’s read range.

As expected, it is observed in the Figure 9, that the reading distance increases when the temperature is increasing since the impedance matching improves. For the operating frequency equal to 800 MHz, when the temperature varies from 25 to 70 °C, the reading distance varies from 2.6 to 4.3 m. On the other hand, when the operating frequency is 866 MHz, the reading distance varies from 4.3 to 5.3 m. It can also be seen that the reading distance range is greater with the 800 MHz frequency than with the 866 MHz frequency, which reflects the better resolution obtained for the sensor.

Consequently, in sensor mode, this study shows the limits of use in terms of minimum reading distance to use the sensor. At full scale (here from 25 to 70 °C), the distance between reader and tag should not exceed 2.6 m at the 800 MHz frequency and 4.3 m at the 866 MHz frequency; otherwise the tag will not be read for the minimum temperature equal to 25 °C. From this point of view, the performance is better for the 866 MHz frequency. Ideally, two opposing criteria should be maximized: on the one hand, maximizing the reading distance for the most unsuitable case (here, a temperature equal to 25 °C), and on the other hand, maximizing the reading distance range when the temperature varies to obtain a better resolution for the sensor.

### 5.3. Analysis of the Temperature RFID Sensor Integrated in a Textile Yarn and Comparison with the Existing Solutions Presented by the Literature

Table 2 presents the properties of wearable UHF RFID temperature sensor tags existing in the literature [9,10,27]. In each work, the sensor is integrated into the RFID chip that can operate in a passive mode but with a degraded performance or in semi-passive mode (integrated battery). The proposed solution allows maintaining the same manufacturing process than the E-Thread^®^ RFID yarn with the advantage of a slender form factor that simplifies its integration in any object and, more importantly, the passive property of the tag is conserved.

## 6. Conclusions and Future Work

This paper presents an RFID temperature sensor tag integrated into a textile yarn. The operation principle of the sensor tag is based on the impedance mismatching due to variation of the resistance of a thermistor as function of temperature. The RFID temperature sensor yarn was fabricated and experimentally characterized. The results validate the proposed approach by the variation of the activation power due to the temperature change. Moreover, a method to avoid a calibration phase before the measurement has been proposed and consists in using a reference tag by assuming that it is subjected to the same temperature change and has the same propagation channel. This assumption allows the calculation of a differential power, which is directly proportional to the temperature variation. 

The results show that in the European UHF RFID band (866 MHz), the temperature sensor tag has the potential to detect a temperature variation of 33.3 °C with 1 dB of differential power. Thus, the sensor tag is more suitable for temperature threshold detection. However, it has been observed that at a frequency below resonance (i.e., 800 MHz), it is possible to detect a temperature variation of 11.4 °C with 1 dB of differential power. In the future, it would be suitable to obtain a similar sensitivity for the sensor tag in UHF RFID band. One solution would be to shift the resonant frequency to higher frequencies, which will require higher activation power levels and ultimately increase the sensitivity of the sensor tag. In the other hand, for an application scenario that does not implies the use of two tags, it could be interesting to exploit a temperature range where the sensor tag exhibits a linear behavior with a fixed temperature curve slope. Indeed, a calibration step would be necessary when the environment is changed, however, around only a single temperature point. Thus, the complete data can be deduced simply by the shift of the original curve of temperature versus the transmitted threshold power, obtained initially in a given environment. 

Finally, it is important to notice that the proposed UHF RFID temperature sensor has a great potential to be used in medical applications where it can provide a wearer’s temperature level. However, the performance of the RFID tag, especially in terms of impedance matching, can be degraded in the presence of the human body. In our case, the impact will be applied on both the reference tag and the sensor tag and can then degrade the reading sensitivity range. In this case, it is possible to ensure a certain robustness by considering from the design stage constraints related to the environment. Presently, we have assumed free space but it will be possible to add a human body model (even a simple one modeled by several layers of superimposed substrates to anticipate the impact). This does not call into question the concept proposed in our study.

## Figures and Tables

**Table 1:** Dimensions of the RFID temperature sensor tag.

Parameter	Dimension (mm)
$L_{1}$	135
$L_{2}$	23
s	0.44
a	0.06
$a^{\prime }$	0.06

**Table 2:** Comparison between the properties of the UHF RFID temperature sensor tag integrated in a textile yarn with solutions existing in the literature.

Reference	Fabrication Method	Substrate	Sensing Principle	UHF Band
[10]	Screen printing	Plastic	RFID chip integrated sensor SL900A	US: 902–928 MHz
[9]	3D printing	Flexible polyimide	RFID chip integrated sensor SL900A	US: 902–928 MHz
[27]	3D printing	Polylactic	RFID chip integrated sensor EM4325	UE: 864.5−867.5 MHz
Current work	E-Thread^®^	No substrate	Tag properties (impedance matching)	UE: 864.5−867.5 MHz
